# Supplementary material for: Genome Editing Using Cas9 Ribonucleoprotein Is Effective for Introducing PDGFRA Variant in Cultured Human Glioblastoma Cell Lines
Source: Int J Mol Sci. 2022 Dec 28;24(1):500. doi: 10.3390/ijms24010500 (PMC9820287; doi:10.3390/ijms24010500)
Supplement: Supplementary file 1 [file ijms-24-00500-s001.zip › ijms-2075992-supplementary.pdf]

A

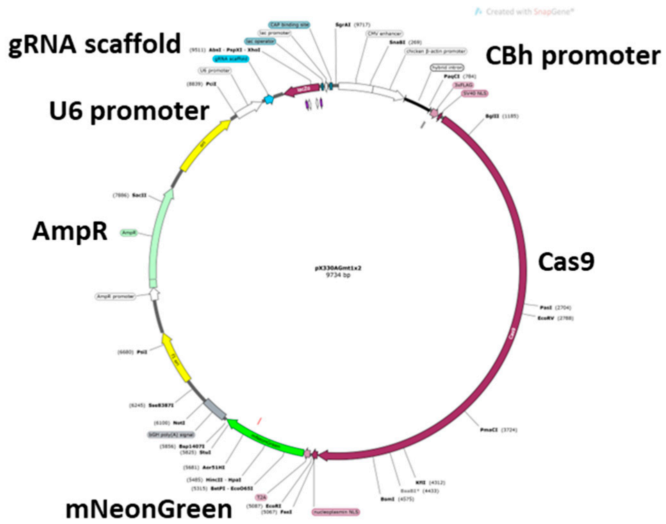

B

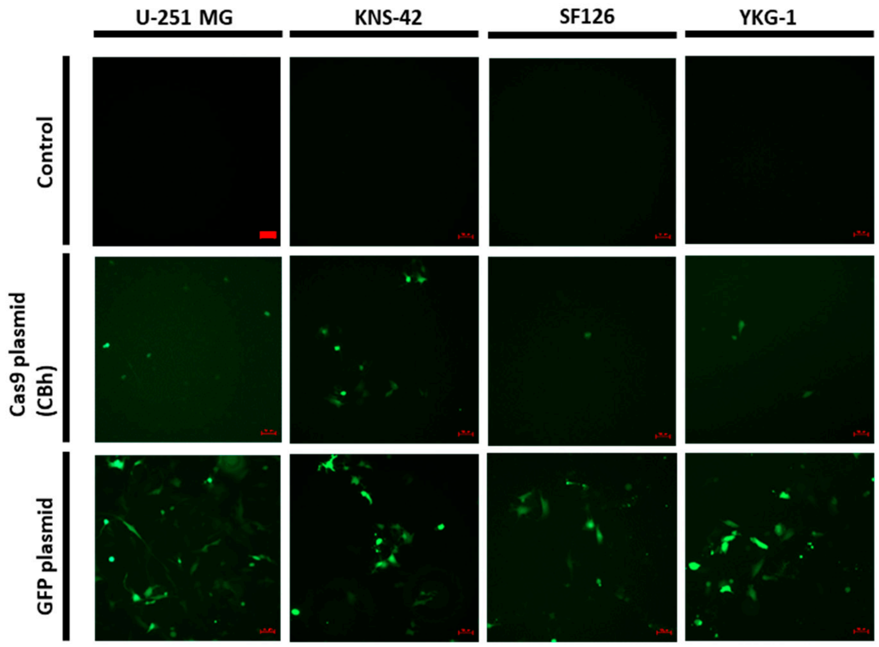

Supplementary Figure S1 Vector map of Cas9 expression plasmid (pX330AGmt-1x2) and fluorescent images captured 72 h after lipofection in GBM cell lines.

(A) Vector map of Cas9 expression plasmid using in this study. (B) Lipofection efficiency (shown in Figure 2A) was calculated using the fluorescence intensity of mNeonGreen and GFP as analyzed using a flow cytometer. Scale bar: 50 µm.

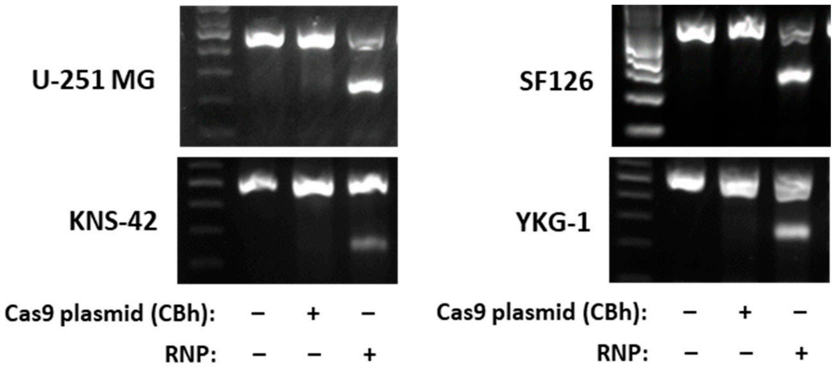

Supplementary Figure S2 Agarose gel images from the T7 endonuclease I assay in GBM cell lines.

The cleavage activity (%, indels), as shown in Figure 2B, was calculated from the intensity of bands analyzed using a densitometer.

A

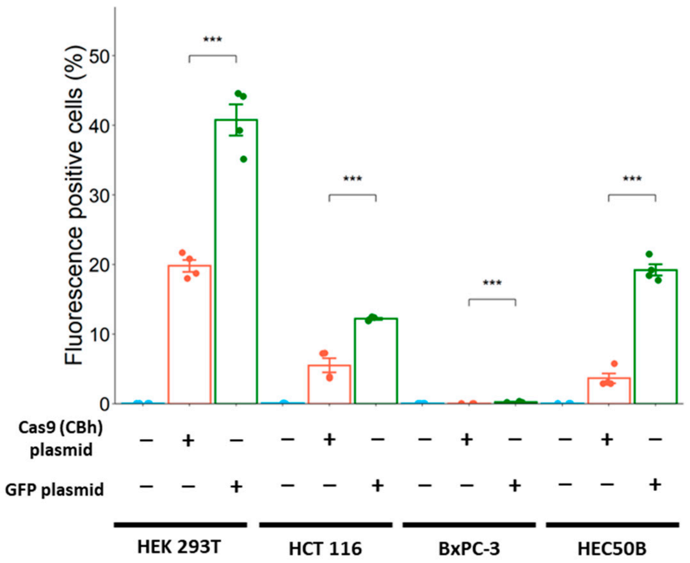

B

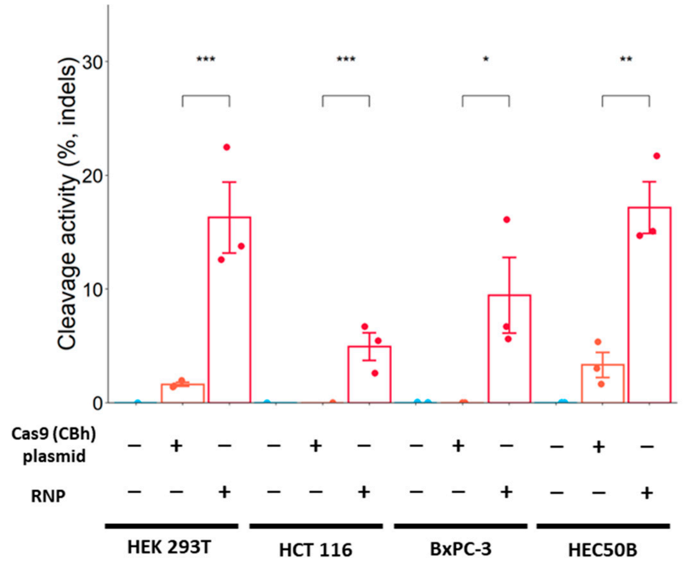

Supplementary Figure S3 Transfection efficiency (A) and cleavage activity (B) in various cell lines.

The transfection efficiency is indicated by the fluorescence intensity of mNeonGreen and GFP. Cleavage activity (indel) was monitored by T7E1 assay and was determined by densitometric analysis of electrophoresis. *$p < 0.05$, **$p < 0.01$ and ***$p < 0.001$ vs. Cas9 (CBh) plasmid.

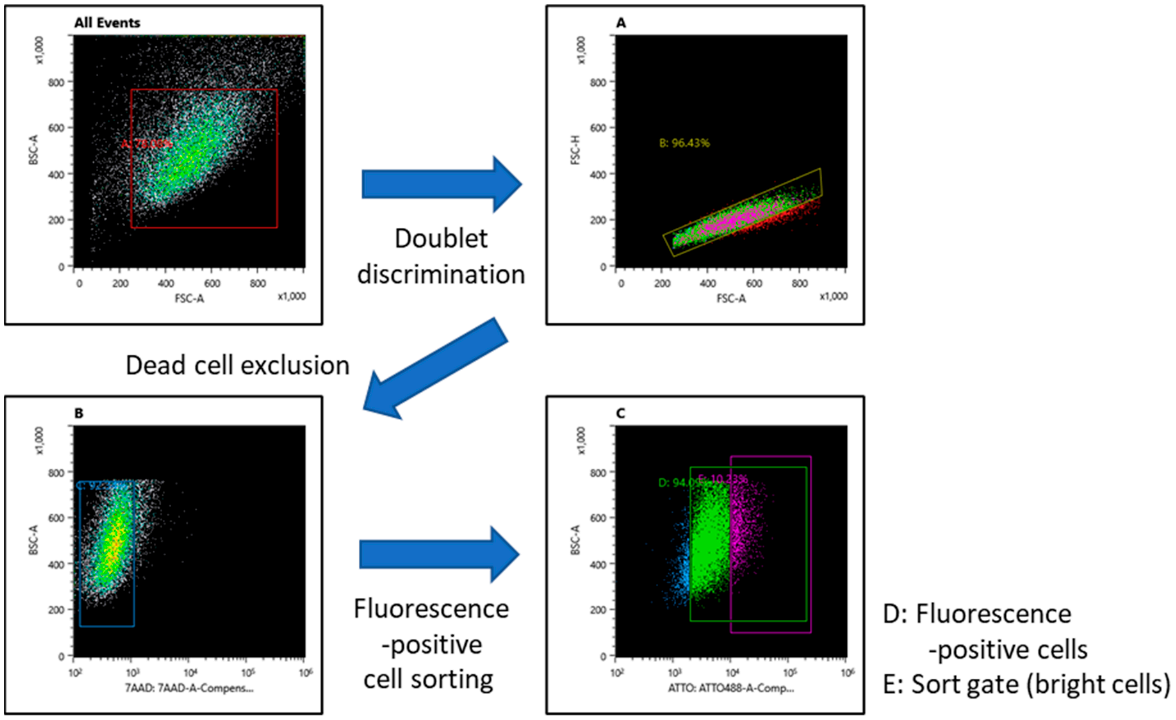

Supplementary Figure S4 Gating strategies used for cell sorting.

Cells were distinguished as populations based on forward-scatter and side-scatter properties. Doublet discrimination and dead cell exclusion were then performed using 7-aminoactinomycin D (7-AAD). Subsequently, cells with high fluorescent intensity were sorted.

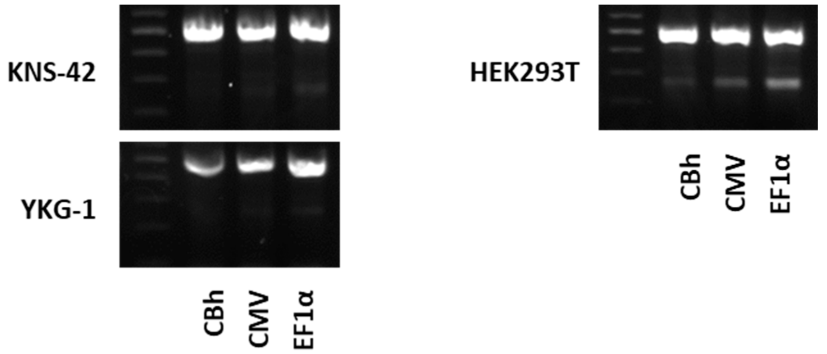

Supplementary Figure S5 Agarose gel images from the T7 endonuclease I assay in KNS-42, YKG-1, and HEK293T cells.

The cleavage activity (%, indels), shown in Figure 4B, was calculated from the intensity of bands as analyzed using a densitometer.

PDGFRα

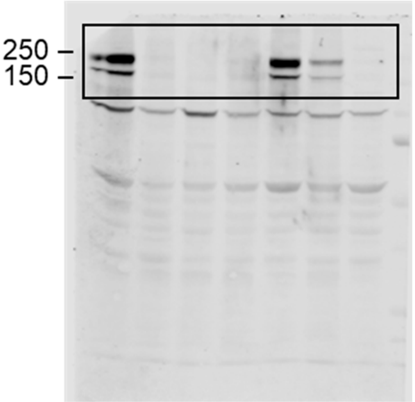

PDGFRβ

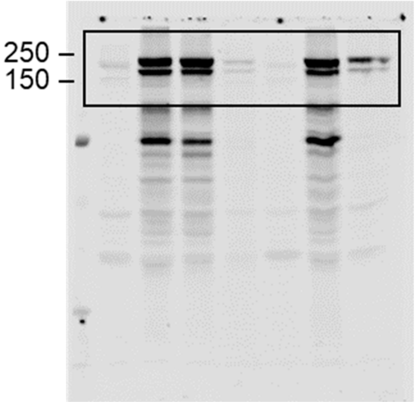

β-actin

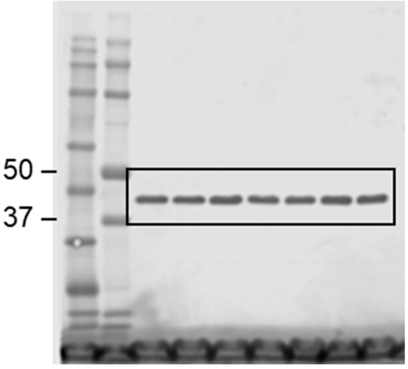

Supplementary Figure S6 Uncropped blots related to Figure 1B.

Cas9

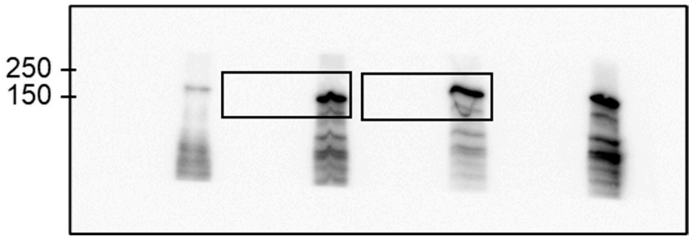

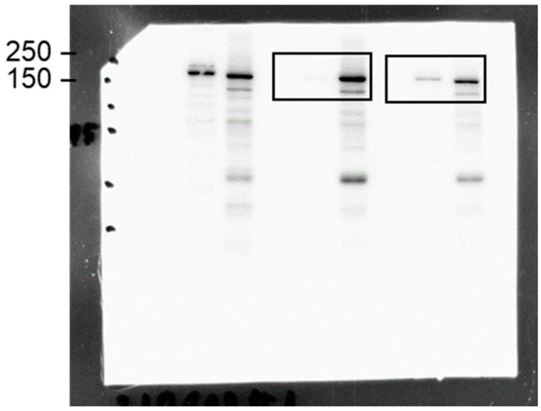

β-actin

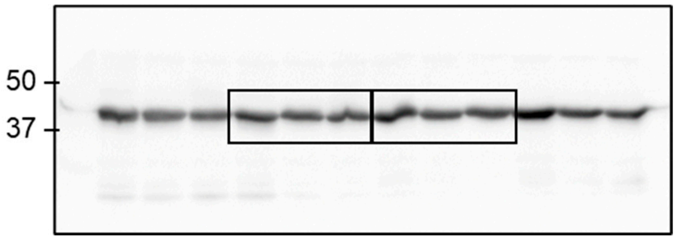

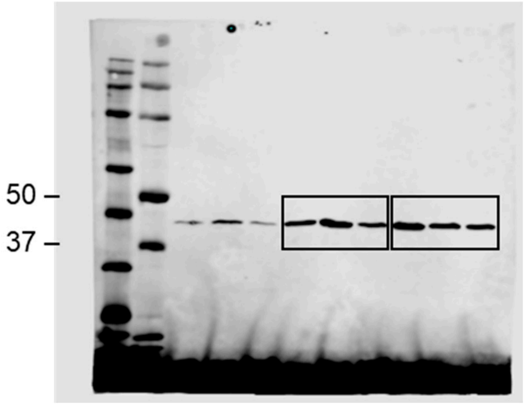

Supplementary Figure S7 Uncropped blots related to Figure 2C.

U-251 MG

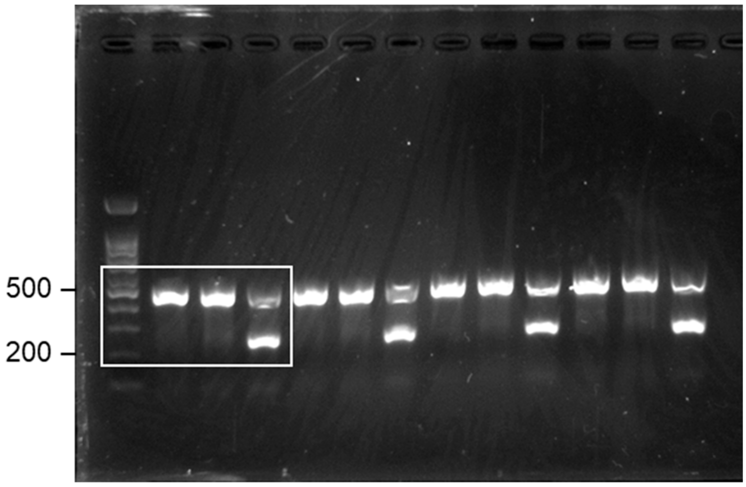

SF126

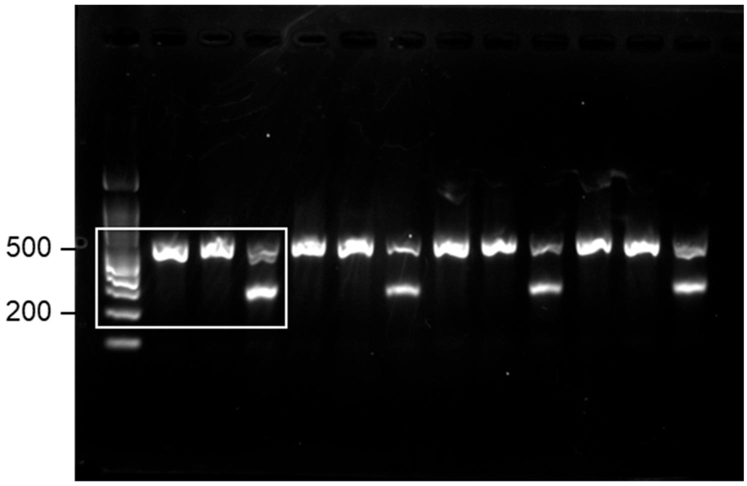

KNS-42

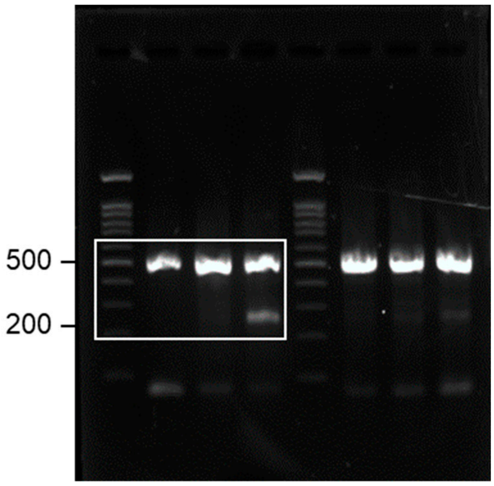

YKG-1

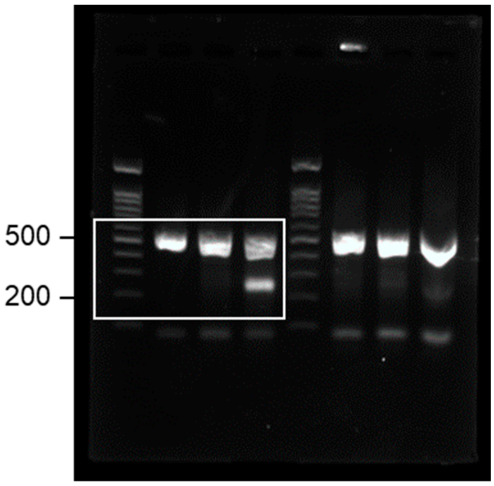

Supplementary Figure S8 Uncropped images related to Supplementary Figure S2.

KNS-42

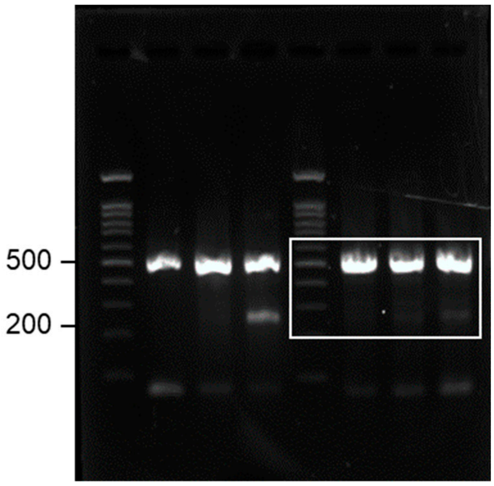

HEK-293T

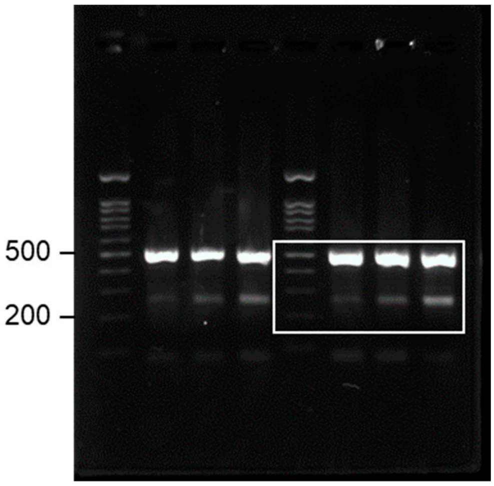

YKG-1

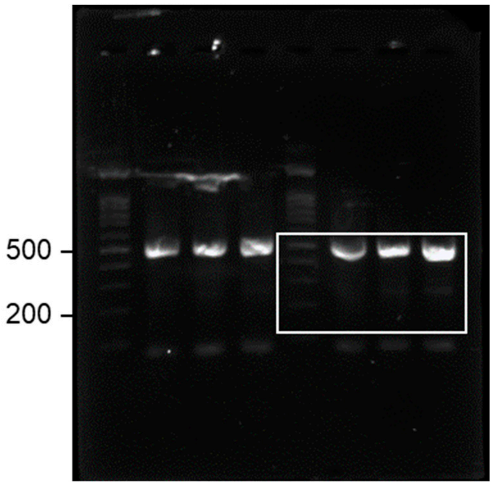

Supplementary Figure S7 Uncropped images related to Supplementary Figure S5.

Supplementary Table S1 Cell lines used in this study.

Cell lines	Media	Fetal bovine serum concentration (%)	Source
U-251 MG	MEM	10	JCRB
T98G	MEM	10	JCRB
A172	DMEM	10	JCRB
NMC-G1	DMEM	10	JCRB
KNS-42	MEM	5	JCRB
SF-126	MEM	10	JCRB
YKG-1	DMEM	10	JCRB
293T	DMEM	10	BRC
HCT 116	McCoy's 5A	10	ATCC
BxPC-3	RPMI-1640	10	ATCC
HEC50B	MEM	15	JCRB

All media were supplemented with 2 mM glutamine, 100 U/mL penicillin, 100 μg/mL streptomycin (Thermo Fisher Scientific, Waltham, MA, USA), and 5%–15% fetal bovine serum (Corning, Corning, NY, USA). Abbreviations: DMEM: Dulbecco's modified Eagle medium (FUJIFILM Wako Pure Chemical, Osaka, Japan); MEM: Eagle's minimal essential medium (FUJIFILM Wako); McCoy's 5A: McCoy's 5A medium (Thermo Fisher Scientific); RPMI-1640: RPMI-1640 medium (Nissui Pharmaceutical, Tokyo, Japan); BRC: Riken BioResource Research Center (Ibaraki, Japan); JCRB: Japanese Collection of Research Bioresources Cell Bank (Osaka, Japan); ATCC: American Type Culture Collection (Manassas, VA, USA).

Supplementary Table S2 Sequences of primers used in this study.

Primers	Sequence (5′–3′)
pX330A-1x2 plasmid modification	
pX330_bone_F	ctagagctcgctgatcagcctcgactgtgccttct
pX330_bone_R	ccctctccactgccgaattcctttttctttttgcctggccggccttttcgtggccgccg gcctttgtcgcctcccagctgagacaggtcg
pX330_mng_F	ggctgatcagcgagctctagttacttgtacagctcgtccatgcccatc
pX330_mng_R	gaattcggcagtggagagggcagaggaagtctgctaacatgcggtgacgtcgag gagaatcctggcccaatggtgagcaagggcgaggaggata
mNGmt_F	aAacttaccccaacgacaaaaccatcatc
mNGmt_R	tcttcgacctgcaccagtccg
sgRNA4_PDGFRA_top	caccgATTGTTGGCCAAAATAGTCC
sgRNA4_PDGFRA_bottom	aaacGGACTATTTTGGCCAACAATc
Promoter exchange in pX330AGmt-1x2	
pXAGmt_F	gttggaccggtgccaccatggactataaggaccac
pXAGmt_R	ggtacctctagaagagaccaccggtggaaagcggg
pXAGmt_CMV_F	tcttctagaggtaccgacattgattattgactagt
pXAGmt_CMV_R	tggcaccggtccaactgactcgaactcgcttccgt
pXAGmt_EF1a_F	tcttctagaggtaccggctccggtgcccgtcagtg
pXAGmt_EF1a_R	tggcaccggtccaactcacgacacctgaaatggaa
T7E1 assay	
KI-F	gctatgtaaacacttagctttcagttgttcatgtg
KI-R	ttctatataacccctatctgggattcaaagttcc
Next-generation sequencing	
gDNA_F	aggaattggccctatacttagg
PDGFRA_e10_R	cagcttcagctctcggttct

Supplementary Table S3 Poring-pulse conditions for electroporation.

Cell lines	Transfer	Voltage (V)	Length (ms)	Intervals (ms)	Polarity
U-251 MG	RNP	150	7.5	50	+
	Plasmid	150	2.5		
KNS-42	RNP	150	7.5		
	Plasmid	150	2.5		
SF126	RNP	150	5		
	Plasmid	150	2.5		
YKG-1	RNP	175	7.5		
	Plasmid	150	2.5		
293T	RNP	150	7.5		
	Plasmid	125	5		
HCT-116	RNP	150	7.5		
BxPC-3	RNP	150	7.5		
HEC50B	RNP	150	5		
	Plasmid	150	2.5		
